# Physical and mental health impact of the COVID-19 pandemic at first year in a Spanish adult cohort

**DOI:** 10.1038/s41598-023-28336-2

**Published:** 2023-03-20

**Authors:** Pere Castellvi Obiols, Andrea Miranda-Mendizabal, Silvia Recoder, Ester Calbo Sebastian, Marc Casajuana-Closas, David Leiva, Rumen Manolov, Nuria Matilla-Santander, Isaac Lloveras-Bernat, Carlos G. Forero

**Affiliations:** 1 Department of Medicine, School of Medicine, International University of Catalonia (UIC), Campus Sant Cugat, Josep Trueta s/n, 08195 Sant Cugat del Vallès, Spain; 2 Department of Basic Sciences, International University of Catalonia (UIC), Sant Cugat del Vallès, Spain; 3 Servei Català de la Salut, Barcelona, Spain; 4 Institut Universitari de Investigació en Atenció Primaria Jordi Gol (IDIAP Jordi Gol), Barcelona, Spain; 5 Department of Social Psychology and Quantitative Psychology, University of Barcelona (UB), Barcelona, Spain; 6 Unit of Occupational Medicine, The Institute of Environmental Medicine (IMM), Karolinska Institutet, Stockholm, Sweden

**Keywords:** Psychology, Infectious diseases, Psychiatric disorders

## Abstract

The COVID-19 pandemic and the political and health measures have profoundly affected the health of our populations. However, very few studies have been published assessing its impact using a prospective cohort. The aim of this study is to describe the impact on physical and mental health due to the COVID-19 pandemic in the general population in Spain, and according to COVID-19 clinical status, during the first year of the pandemic. A longitudinal cohort study with two online surveys were performed on a representative sample of the adult Spanish population before (N = 2005, October/November 2019) and during the pandemic (N = 1357, November/December 2020). We assessed disability using the World Health Organisation Disability Assessment Schedule (WHODAS), major depressive episode (MDE) and suicidal thoughts and behaviours (STB), using an adapted version of the Composite International Diagnostic Interview (CIDI 3.0); generalised anxiety disorder (GAD) using the GAD-7 scale; post-traumatic stress disorder (PTSD) symptoms using the PTSD checklist for DSM-5 (PCL-5). For physical health, there was a statistically significant loss of weight (mean/SD) (T0, 73.22/15.56 vs. T1, 71.21/11.94), less use of tobacco (T0, 11.4% vs. T1, 9.0%) and decreased disability (mean/SD) (T0, 21.52/9.22 vs. T1, 19.03/7.32). For mental health, there was a significant increase in MDE (T0, 6.5% vs. T1, 8.8%) and in the prevalence of GAD (T0, 13.7% vs. T1, 17.7%). The prevalence of STB (T0, 15.1% vs. T1, 7.1%) significantly decreased. Individuals who declared they had been diagnosed with COVID-19 (3.6%) showed a worsening in physical health and an increase in mental health problems and PTSD symptoms. Although suicide risk during the first year of the pandemic was significantly less, many suicide risk factors increased: such as the incidence and persistence of MDE and GAD, the presence of PTSD symptoms in those diagnosed with COVID-19, and a worsening in self-assessed health status. We expect an increase in STB in the population in the long-term. Future research should gather information about the long-term impact of the pandemic.

## Introduction

The COVID-19 pandemic and the political and health measures taken to curb the spread of SARS-CoV-2 have profoundly affected every aspect of day-to-day life^1^. Spain, with its first case on 31 January 2020, is one of the countries in Europe most affected by infections, complications, and deaths^2^. It was not until February 24 when Spain confirmed several new COVID-19 cases related to a recent SARS-CoV-2 outbreak in the north of Italy. Since that date, the number of COVID-19 cases grew exponentially in Spain so that by March 30, there were over 85,199 confirmed cases, and 7424 deaths, according to the official numbers. On March 25, the death toll attributed to COVID-19 in Spain surpassed that of mainland China. The economic and social impact of the COVID-19 pandemic in Spain is without precedent. To combat the pandemic, the Spanish Government implemented a series of social distancing and mobility restriction measures. First, all classes at all educational levels were cancelled in the main hot spots of the disease on March 10. On March 14, the Government of Spain declared a state of emergency for 2 weeks across the entire country closing all schools and university classes, and workers were encouraged to tele-work. Despite these efforts, the daily growth rate in the number of confirmed COVID-19 cases continued to grow. Thus, on March 30, new mobility restriction and social distancing measures were implemented; all nonessential labour activity was to be interrupted for a 2-week period. Moreover, the Spanish Government extended the state of emergency until June 21^2^. Although these interventions put a halt to the normal daily lives of most people in Spain, their impact on people’s economic, physical, and mental well-being were unknown at the time. Many other countries implemented similar measures. Studies show an impact on employment and livelihoods, income, and personal debt^3^, coupled with increased worries about future job insecurity and probable physical and psychological worsening^4–6^.

Since the emergence of the COVID-19 pandemic, substantial efforts have been made to understand its transmission and to assess the socio-economic and health impact of the pandemic, due to the political measures, economic recession, and social crises. Previous literature suggests a link between pandemics and a worsening in physical health, such as an increase in obesity rates^7^, chronic physical symptoms, frailty, coronary heart disease, malnutrition, hospital readmission and early mortality^8^. The lockdown culture, loneliness, socio-economic instability, changes in eating habits and an increase in sedentary, domestic activities might have a further deleterious effect on physical health^9^.

Mixed results have been found regarding the impact of the pandemic on mental health. In Denmark^10^ and the United Kingdom^1^, results from a cohort study showed worsening mental health among the general population. However, the COVID-19 pandemic does not seem to have further increased depressive, anxiety and obsessive-compulsive symptom severity, compared with pre-pandemic levels in The Netherlands^11^. As for Spanish data, the general adult population has mostly reported an increase of depressive^12^ and anxiety symptoms following the immediate consequences of the first wave of the COVID-19 pandemic (spring 2020). However, these previous studies in Spain used a retrospective cross-sectional design. In an earlier published article using a longitudinal, population-based cohort study of Spaniards just after the first wave of infection (spring/summer 2020), the prevalence of depression and suicidal ideation were not significantly increased^13^. However, there is a need to know the medium- and long-term mental health impact of the COVID-19 pandemic on the population, using a prospective, longitudinal design study, with assessments before and during the pandemic. Through this a prospective cohort, it allows us to measure changes of some physical and mental health outcomes in the Spanish population and uncover temporality, which is one of the postulates about causality, making comparisons with prepandemic data, and evaluate changes in the health of our population.

The aim of this study is to describe the impact on physical and mental health due to the COVID-19 pandemic in the general population in Spain, and according to COVID-19 clinical status, during the first year of the pandemic.

## Methods

### Study design

A longitudinal cohort study, with two assessments from an online survey of Spanish residents was carried out. The baseline reference survey (T0) was acquired as part of the BIOVAL-D study (ISCII-FEDER Exp: PI16/00165) during October/November 2019 and assessed mental health prior to the SARS-CoV-2 outbreak. The follow-up survey (T1) was conducted after 12 months (November/December 2020) using the same questionnaire and adding some extra dimensions and variables to identify physical and mental health outcomes, their risk and associated protective factors during the SARS-CoV-2 outbreak during and after lockdown, using clinical characteristics of people diagnosed with COVID-19. The survey had an approximate duration of 30 min. This study was approved by the Ethical Committee of the institution (Reg. No.: MED-2020-02) and has been performed in accordance with the Declaration of Helsinki. An informed consent form was signed by each participant using an online version. Data were recorded in a centralized database and anonymized before statistical analysis and shared for all authors.

### Study sample

A Spanish, nationally representative, population-based sample (≥ 18 years old) was chosen, representative of geographical, sex, age and socioeconomic status. At the baseline assessment (T0), participants were recruited from a secure online panel data vendor, resulting in a final sample of 2005 individuals. For the follow-up survey, all 2005 participants were contacted by the panel provider and invited to participate. Participants received an informative email on the study objectives and characteristics, including a link to an informed consent form which acted as a filter for entering the survey. Baseline participants were offered participation in the follow-up and those who did not sign consent forms or did not fully fill in the survey were excluded.

From the initial 2005 individuals, 941 participants answered the follow-up survey; a participation rate of 46.9%. To ensure representativeness of the post-pandemic sample, an additional representative panel was invited to participate. Participants from the additional panel who were invited to participate at the follow-up were matched by sex, geographical residence and age, to ensure similar characteristics to the baseline participants who did not respond to the follow-up. From this additional panel, 416 participants were recruited; giving a total of 1357 participants included in the analysis at 12 months.

### Variables

#### Socio-demographic variables (T0)

Age, sex, marital and employment status were recorded. Age was a continuous variable; Sex had two response options Male and Female; Marital status was recorded as Single, Married or Living with a couple, Separated, Divorced and Widowed; and, finally, Employment status as Employed, Off sick, Unemployed, Homemaker, Student, Both student & employed, Temporal or permanent disability, and Retired. These socio-demographic variables were considered as nominal.

#### Health status (T0 & T1)

Physical and mental health and smoking status were self-assessed. The short version of the World Health Organisation Disability Assessment Schedule (WHODAS 2.0, 12 items) was used for assessing disability, and it is recommended for epidemiological studies. The WHODAS 2.0 showed have excellent internal consistency in all languages (alpha ≥ 0.90)^14^.This is a 12-item, self-administered scale. Items are grouped by pairs in 6 domains: Understanding and communicating with the world; Moving and getting around; Self-care; Getting along with people; Activities of daily living (domestic responsibilities, leisure, and work); and Participation in society. Scoring is on a range of 12–60, where 12 means no disability and 60 the highest disability. Response options were using a Likert Scale (None = 1; Mild = 2; Moderate = 3; Severe = 4; Extreme or cannot do = 5). The scale has been validated for the Spanish population^15^. Internal consistency in our sample was good in both assessments (baseline, alpha = 0.93; follow-up, alpha = 0.87). The distribution of disability using WHODAS 2.0 ranged from 12 to 58 at T0, and from 12 to 49 at T1, showing a normal distribution. This variable was considered as a continuous.

Physical and mental health self-assessment and reported health transition one year ago were provided using The MOS 36-Item Short-Form Health Survey (SF-36)^16^. The SF-36 is a widely used and patient-reported measure of health status. Physical and mental health were assessed using 2 items with 5 response options (Excellent; Very Good; Good; Fair; Poor). Health transition response options were: Much better; Somewhat better now; About the same; Somewhat worse now; Much worse). These variables were considered as an ordinal. The Spanish version of the SF-36 has been used^17^. Self-reported anthropometric measurements relating to body mass index (BMI) were collected. BMI was recorded as an ordinal scale which: < 18.5 kg/m^2^: underweight; 18.5–24.9 kg/m^2^: normal; 25–29.9 kg/m^2^: overweight; and ≥ 30 kg/m^2^: obese.

#### COVID-19 exposure (T1)

Items about COVID-19 exposure were developed ad hoc for this study. Data about having been tested for or diagnosed with COVID-19 were gathered, including related symptoms. Items developed were “Have you ever been tested for COVID-19?”, response options were “Yes/No/No, although I had related symptoms, I was not tested”. If the subject answered Yes, then an additional question was administered “Was this test positive?”. COVID-19 clinical status was classified into 4 groups: Group 1, individuals with no COVID-19 symptoms and no COVID-19 test done; Group 2, those with COVID-19 symptoms with no test done; Group 3, those with COVID-19 test done with a negative result; and Group 4, those with COVID-19 test done with a positive result. Related symptoms administered in case an individual responded “Yes” or “No, although I had related symptoms, I was not tested” were: (a) Cough; (b) Fever; (c) Difficulty breathing or shortness of breath; (d) Sore throat when you drink any liquid; (e) Loss of smell; (f) Loss of taste; (g) Muscle aches; (h) Diarrhoea; (i) Chest pain; (j) Headache; (k) Coughing up blood; (l) Vomiting; (m) Feeling confused; (n) Feeling drowsy; (o) Feeling very tired; (p) Had other related symptoms; (q) Didn't have any symptoms.

Information about the number of friends and relatives infected with COVID-19 and their mortality were also assessed using a continuous variable. Finally, stress related to the COVID-19 outbreak and its possible effects (e.g., family finances, increased social isolation and worry about getting infected) were also evaluated using six items using a Likert scale with five response options: Not at all; A bit; Quite; A lot of; Very much. Cronbach’s alpha of stress related to COVID-19 in our sample was 0.83 showing good internal consistency. For some analyses, COVID-19 clinical status was collapsed merging Group 1 and Group 3 versus Group 2 and Group 4 due to the small number of individuals in some groups.

#### Mental health

##### Major depressive episode (T0 & T1)

For the assessment of Major Depressive Episode (MDE), the full screening section (8 items) from the Composite International Diagnostic Interview (CIDI) version 3.0 was used^18^. This section works as a filter to enter the diagnosis section, meaning that only those who answer some of the items positively go on to answer following questions. The diagnostic section includes 37 items divided into 8 sections: depression and anhedonia (6 items); weight (5items); insomnia (5 items); retardation and agitation (4 items); fatigue (2 items); worthlessness and guilt (5 items); concentration (4 items); suicide (6 items) that evaluate the presence or absence of MDE symptoms for at least two weeks. When five criteria were achieved, individuals must have a high grade of disability of > 50 in WHODAS to establish the diagnosis^19^. The area under the curve was 0.75^20^. The CIDI has been translated into many languages, including Spanish^18^. The prevalence at 12 months was assessed at T0, and since the lockdown started (March 14) at T1.

##### Generalised anxiety disorder (T0 & T1)

The Generalised Anxiety Disorder-7 scale (GAD-7) was administered, which consists of 7 items answered with a 4-point Likert scale and total scores ranging from 0 to 21. Point prevalence (2 weeks) was assessed at T0, and since the lockdown started (March 14) at T1. For the Spanish version, Cronbach's alpha coefficient of 0.93 was obtained. Taking into account the 10-point cut-off, sensitivity values of 86.8% and specificity of 93.4% were found^21^. To establish a diagnosis, individuals must also have a high degree of disability of > 50 in WHODAS^19^. The GAD-7 was administered to all those with positive depression screening using CIDI instrument and, additionally, a randomized 40% with negative screening of the baseline sample (n = 722) and the entire sample at the follow-up assessment (n = 1357).

##### Symptoms of post-traumatic stress disorder (PTSD) (T1)

To assess DSM-5 symptoms of PTSD related to the experience of being infected with COVID-19 or the death of somebody close due to COVID-19, an adapted Spanish version of the PTSD Checklist for DSM-5 (PCL-5) was used (20 items). Responses to each item are rated using a 5-point scale, ranging from 1 (Not at all) to 5 (Extremely), and indicating the extent to which respondents had been bothered by that symptom in the past 7 days. Scoring ranges from 20 to 100. The higher the score, the more symptoms of PTSD. The PCL-5 demonstrated that the scale had solid psychometric properties (alpha = 0.97; ICC = 0.96; and convergent validity with other PTSD symptom scales)^22^. The differential item functioning of the PCL-5 scale score indicated that the Spanish version is equivalent to the original language^23^. The PCL-5 was adapted ad hoc in the context of being exposed to the COVID-19 pandemic (e.g. …*avoid memories, thoughts or feelings related to being infected or someone has died from COVID-19*). The PCL-5 was administered to all those with positive COVID-19 test or with any relative or someone known infected by COVID-19 and, additionally, a randomized 20% of the rest of the follow-up sample (n = 720).

The PCL-5 was administered to all those who underwent a COVID-19 diagnostic test, all those who knew a person who died from COVID-19 and a randomised selection of 20% of the rest of the sample. Cronbach’s alpha in our sample was 0.96 showing good internal consistency. The distribution of the PCL-5 was skewed being against the null hypothesis that it is normally distributed (median/Q1-Q3) (14/7–34).

##### Suicidal thoughts and behaviours (STB) (T0 & T1)

The STB^24^ was assessed for ideation, plan or attempt with a single item for any symptom (total 4 items) from the CIDI questionnaire. Suicidal ideation was classified as passive “Did you ever think it would be better if you were dead?” and active ideation “Have you ever thought about killing yourself?”; suicidal plan with “Did you make any plans to harm or kill yourself?”; and suicide attempt with “Did you try to harm yourself or attempt suicide?”. Response items were “Yes/No/I don’t know”. 12 months prevalence was assessed at T0, and since the lockdown started (March 14) at T1.

MDE, GAD and STB were recorded as follows: No mental health problem (negative in T0 and T1), Incidence (negative in T0 but positive in T1), Persistence (positive in both assessments T0 and T1) and Recovery (positive in T0 and negative in T1). These variables were considered as a dichotomous (Yes/No).

##### Sample size

Sample size was estimated based on the incidence data between T0 and T1, assuming an annual baseline depression incidence of 2% and COVID-19 exposure affecting 10% of the population, increasing incidence up to 10%. Based on these assumptions, with a statistical power of 0.80 at a 0.05 nominal significance level and considering a 40% loss-to-follow up cases, total sample size at follow-up was estimated in 1200 people. Depression incidence was selected for this purpose because it was expected to be especially high in the pandemic context being a good proxy variable for mental health effects. Furthermore, it has been done to be consistent with the criteria used in the baseline study, ensuring comparability.

### Statistical analysis

Statistical analyses involve different methods depending on the use of cross-sectional or longitudinal data. In cross-sectional data, the prevalence and mean (plus standard deviation) or median (plus the interquartile range between quartiles 1 and 3) of socio-demographic characteristics, COVID-19 exposure, and physical and mental health were calculated. Prevalence at TO was conducted with 2005 individuals and T1 with 1357 individuals. Longitudinal data analyses were conducted for assessing trajectories before and during the pandemic in physical health and mental health problems. Longitudinal analyses to assess changes of mental health status during the first year of follow-up were conducted with 941 individuals. Due to its online nature, cross-sectional data contained no missing data other than interview skips by design. For the missing values lost to follow up, we corrected using inverse probability weighting (IPW)^25^, calculated as the inverse of the logistic propensity of completing the follow-up survey, conditioned on observed related covariates. Population weights were applied to restore sex, geographical and age population distribution.

McNemar’s test was used to assess changes in the sample between T0 and T1 in categorical variables; the Student’s paired samples *t*-test was used in continuous variables for assessing mean differences across time.

Physical and mental health problems were assessed for their association with COVID-19 clinical status in 2 groups of the COVID-19 clinical status (positive or those with no test done but COVID-19 symptoms vs. Negative test result or No test done and no COVID-19 symptoms) with Chi-squared (*χ*^2^) and Cramer’s V (*V*_*c*_). Finally, group differences between the level of disability and COVID-19 was assessed with the Student’s parametric t-test and, finally, PTSD symptoms and COVID-19 clinical status was assessed with the U-Mann non-parametric test for independent samples because most of individuals had no or few symptoms of PTSD not supporting visually and statistically the hypothesis of normality (Kolmogorov–Smirnov normality test *p* < 0.001). Some independent variables of physical and mental health were collapsed because very few individuals were found in some subgroups.

Due to the low number of individuals in some comparisons, a sensitivity analysis was done between physical and mental health outcomes after collapsing for increasing the number of individuals in each group and COVID-19 clinical status (Supplementary Table S2).

All statistical tests were conducted with R package *ipw*^26^ and SPSS v20.0. Significance level was corrected for multiple testing with False Discovery Rates (FDR) method using the Benjamini–Hochberg adjustments^27^, with a significance level of 5%.

### Data availability

The study protocol and individual participant data that underlie the results reported in this article, after de-identification, can be shared with investigators whose proposed use of the data has been approved by the ethic committee of the Universitat Internacional de Catalunya (UIC). Data can be provided for meta-analysis or other projects. Requests should be addressed to the senior author at pcastellvi@uic.es.

### Ethics approval

The Ethical Committee of the Universitat Internacional de Catalunya approved the follow-up study. The previous study was approved by the Ethical Committee of the IMIM-Parc de Salut Mar.

## Results

### Prevalence of physical and mental health before (T0) and during (T1) the COVID-19 pandemic

Attrition analyses identified differences among individuals who responded to the T1 subsample as compared to the baseline sample (T0) in gender and age range, but not in the Spanish regions (see Supplementary Table S1). The follow-up sample had a lower percentage of men (T0, 51.1% vs. T1, 44%), and a higher percentage of older people (> 65: T0, 14.2% vs. T1, 20.8%) than the baseline sample.

Baseline characteristics of the whole T0 sample and the follow-up T1 subsample are shown in Table 1. Table 1 summarises the weighted characteristics of the sample, 48.5% were men, 53.8% of the sample had an age range of 40–65 years, 31.3% were single and 56.6% were married, more than half of the sample were employed (54.6%) and 20.4% were retired.

**Table 1:** Comparison of sample characteristics at baseline and 12-month follow-up samples after weighting.

	Baseline N = 2005		12-month follow-up N = 1357		
	N	%	N	%	p*
Socio-demographic characteristics					
Gender, *(men)*	969	48.5			
Age, (years)					
18–25	125	6.3			
> 25–40	444	22.2			
> 40–65	1074	53.8			
> 65	355	17.8			
Marital status					
Single	625	31.3			
Married/Couple	1130	56.6			
Separated	23	1.1			
Divorced	120	6.0			
Widowed	100	5.0			
Employment status					
Employed	1090	54.6			
Off sick	30	1.5			
Unemployed	158	7.9			
Homemaker	139	7.0			
Student	71	3.6			
Student & employed	57	2.8			
Temporal or permanent disability	44	2.2			
Retired	408	20.4			
Physical health					
Self-perception					0.570
Excellent	100	5.0	64	4.7	
Very good	423	21.2	281	20.8	
Good	1127	56.4	745	55.2	
Fair	309	15.4	234	17.3	
Poor	39	2.0	26	2.0	
Current general health self-perception than					**0.002**
before the pandemic	81	4.0	30	2.2	
Much better	253	12.7	57	4.2	
Somewhat better	1407	70.5	952	70.5	
Same	235	11.8	283	21.0	
Somewhat worse	21	1.1	28	2.1	
Much worse					
Weight (kg) (mean/SD)	73.22	15.56	71.21	11.94	**0.002**
BMI					0.589
Underweight	37	1.9	33	2.4	
Normal	920	46.2	590	43.7	
Overweight	728	36.5	525	38.9	
Obese	307	15.5	195	14.4	
Smoking					**0.002**
Non-smoker	1211	60.6	917	67.9	
Former smoker	433	21.7	251	18.6	
Occasional	126	6.3	61	4.5	
Current	227	11.4	121	9.0	
Disability *(mean/SD)*	21.52	9.22	19.03	7.32	**0.002**
Mental health					
Self-perception					**0.008**
Excellent	310	15.5	159	11.8	
Very good	668	33.5	432	32.0	
Good	887	44.4	616	45.6	
Fair	123	6.2	129	9.5	
Poor	10	0.5	14	1.0	
Major depressive episode (yes)	131	6.5	119	8.8	**0.036**
Generalized anxiety disorder *(yes)*	99	13.7	75	17.7	**0.002**
Posttraumatic stress disorder symptoms (median/ Q_1_-Q_3_*)*			14	7–34	
Thoughts of death					0.079
Yes	480	24.0	378	28.0	
I don’t know	64	3.2	34	2.5	
Any suicidal thoughts and behaviors	294	15.1	9.4	7.1	**0.002**
Suicidal ideation (passive)					**0.002**
Yes	276	13.8	104	11.3	
I don’t know	50	2.5	16	1.8	
Suicidal ideation (active)					**0.002**
Yes	87	4.4	31	2.3	
I don’t know	34	1.7	7	0.5	
Suicidal plan					0.081
Yes	41	2.1	9	0.7	
I don’t know	11	0.6	6	0.4	
Suicide attempt					0.129
Yes	26	1.3	5	0.4	
I don’t know	7	0.3	1	0.1	

*Categorical variables were assessed with McNemnar’s test, and continuous variables with paired t-test. *p*-values were adjusted by multiple comparison with False Discovery Rates (FDR). Statistically significant differences between T0 and T1 were conducted only.

Kg, Kilograms; Q_1_, First quartile_;_ Q_3_, Third quartile; SD, Standard deviation.

% weighted follow-up sample weight (inverse probability weighting and post-stratification).

Significant values are in bold.

Overall, there were no statistically significant differences in the prevalence of self-assessment of physical health during the pandemic compared with before (*p* = 0.532), although more people considered their general health was somewhat or much worse than somewhat or much better during the pandemic, when compared with before the pandemic (*somewhat or much worse*, 23.1% vs. *somewhat or much better*, 6.6%; *p* = 0.002). Additionally, the prevalence of current smokers was statistically significantly lower (T0, 11.4% vs. T1, 9.0%; *p* = 0.002); the population had statistically significant lower weight (mean/SD) (T0, 73.22/15.56 vs. T1, 71.21/11.94; *p* = 0.002); and the WHODAS indicated there was a statistically significant decrease in disability (mean/SD) (T0, 21.52/9.22 vs. T1, 19.03/7.32; *p* = 0.002) during the pandemic.

Regarding self-assessed mental health, a higher prevalence of fair or poor self-assessment was observed during the pandemic than before (T0, 6.7% vs. T1, 10.5%; *p* = 0.002). For mental disorders, there were statistically significant differences in the prevalence of MDE (T0, 6.5% vs. T1, 8.8%; *p* = 0.036) and GAD (T0, 13.7% vs. 17.7%; *p* = 0.002). Finally, we found that the prevalence of any STB (T0, 15.1% vs. T1, 7.1%; *p* = 0.002), and passive (T0, 13.8% vs. T1, 11.3%; *p* = 0.002) and active suicidal ideation (T0, 4.4% vs. T1, 2.3%; *p* = 0.002) were statistically significantly decreased during the pandemic.

We also assessed specific variables related to the COVID-19 pandemic. Results showed that 514 (38.1%) of the total sample received a diagnostic test for COVID-19 and 3.4% reported symptoms related to COVID-19 but they did not undergo any diagnostic test. Of the 514 participants who had a COVID-19 diagnostic test, 48 (9.3%) were positive, which represents 3.6% of the total sample. The most prevalent COVID-19 symptoms were headache (18.8%), cough (16.3%), muscle aches (15.1%), fever (15.0%), feeling very tired (13.7%) and diarrhoea (10.1%). Taking into account the number of people known by participants to be infected, the observed median (Q_1_-Q_3_) was 22(16–33). For people known by participants to have died, the observed median (Q_1_-Q_3_) was 6(5–8). Individuals reported different reasons for being worried: (i) a lot or very much about the increase in social distancing (19.5%); (ii) difficulties getting the help needed for their loved ones (19.8%); and (iii) the probability of their loved ones becoming infected (27.5%) during the pandemic (Table 2).

**Table 2:** Sample characteristics during the COVID-19 pandemic (at 12-month follow-up) of factors associated and stress-related with COVID-19 after weighting.

	12-Month follow-up N = 1357	
	N	%
Factors associated with COVID-19		
COVID-19 clinical status		
Positive test result	48	3.6
Negative test result	466	34.5
COVID-19 symptoms without test done	45	3.4
No test done and no symptoms	791	58.5
COVID-19 symptoms		
Cough	92	16.3
Fever	85	15.0
Difficulty breathing or shortness of breath	47	8.3
Sore throat when you drink any liquid	30	5.2
Loss of smell	53	9.2
Loss of taste	47	8.1
Muscle aches	86	15.1
Diarrhoea	56	10.1
Chest pain	25	4.4
Headache	107	18.8
Coughing up blood	0	0.0
Vomiting	16	2.8
Feeling confused	13	2.3
Feeling drowsy	23	4.2
Feeling very tired	78	13.7
Had other related symptoms	18	3.2
Number of known infected people (median/Q_1_–Q_3_)	22	16–33
Number of known death people by COVID-19 (median/Q_1_–Q_3_)	6	5–8
COVID-19 stress-related		
Financial problems		
Not at all	675	50.0
A bit	376	27.8
Quite	175	13.0
A lot of	67	5.0
Very much	57	4.2
Increase of social isolation		
Not at all	258	19.1
A bit	472	35.0
Quite	357	26.4
A lot of	162	12.0
Very much	101	7.5
Difficulties to get the needed help to our loved ones		
Not at all	267	19.8
A bit	469	34.7
Quite	347	25.7
A lot of	157	11.6
Very much	111	8.2
Have increased arguments with our family and friends		
Not at all	722	53.5
A bit	384	28.4
Quite	157	11.6
A lot of	59	4.4
Very much	29	2.2
The probability to get infected		
Not at all	369	27.3
A bit	503	37.3
Quite	260	19.3
A lot of	129	9.6
Very much	89	6.6
The probability about loved ones getting infected		
Not at all	173	12.8
A bit	413	30.6
Quite	394	29.2
A lot of	208	15.4
Very much	163	12.1

Q_1_, First quartile_;_ Q_3_, Third quartile.

% weighted follow-up sample weight (inverse probability weighting and post-stratification).

### Trajectories of health problems during the COVID-19 pandemic

We found that only 0.5% (*weighted n* = 11) of non-smokers at baseline started smoking at follow-up and 1.4% (*weighted n* = 30) of smokers at baseline quit smoking.

**Figure 1:**
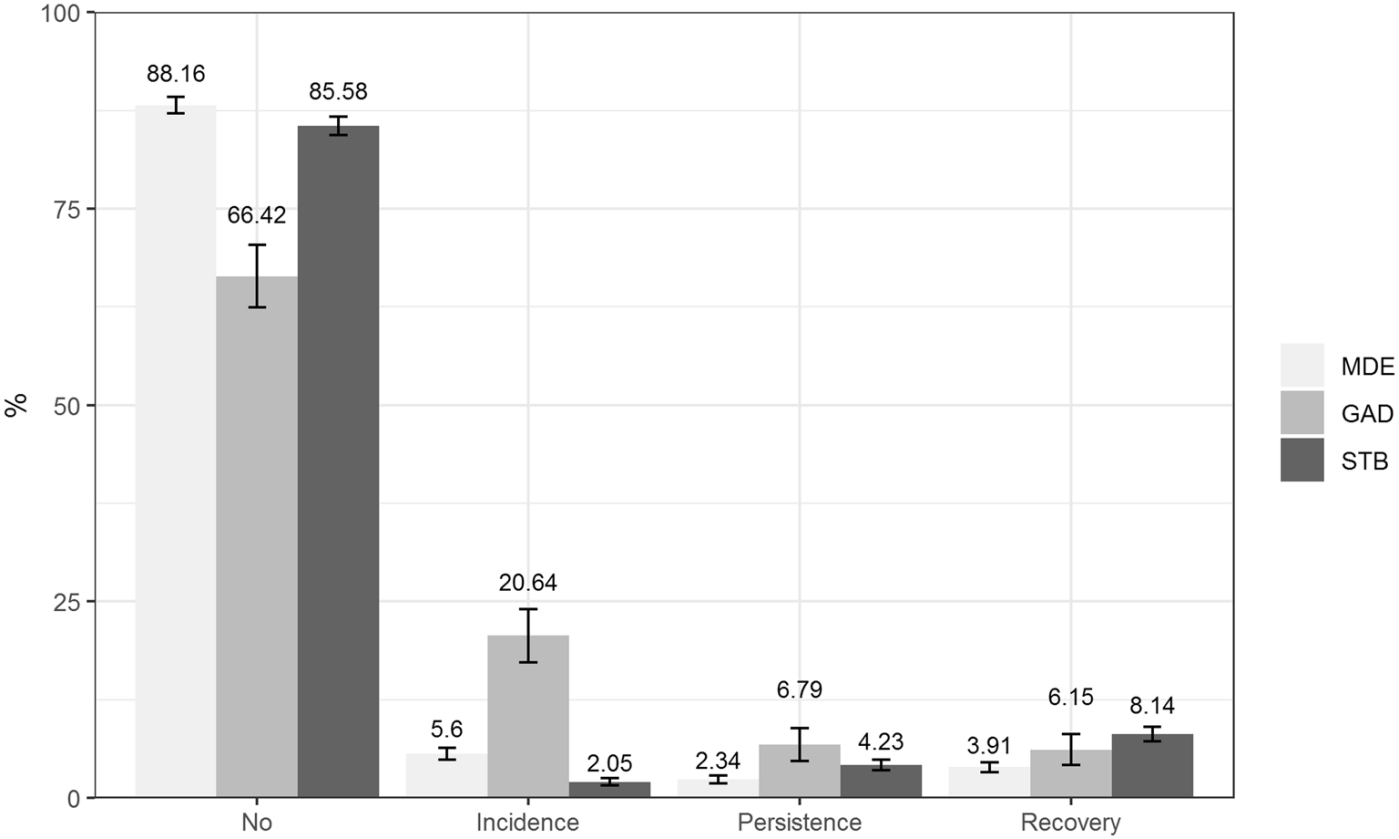
Percentage of sample with No (negative at both assessments T0 and T1), Incidence (negative at T0 but positive at T1), Persistence (positive at both assessments T0 and T1) and Recovery (positive at T0 and negative at T1) of MDE, GAD and any STB before and during the COVID-19. *GAD* Generalized anxiety disorder; *MDE* Major depressive episode; *STB* Suicidal thoughts and behaviors. % weighted follow-up sample weight (inverse probability weighting and post-stratification). Statistical analyses were conducted with 941 individuals.

When we assessed mental health, the highest incidence in our sample was in GAD (20.6%); MDE was less (5.6%) and for any STB was 2.1%. Persistence was also highest in GAD (6.8%), followed by any STB (4.2%) then MDE (2.3%). Finally, the percentage of individuals who recovered from a baseline mental health problem was the highest for any STB (8.1%), followed by GAD (6.2%) and the lowest for MDE (3.9%) (Fig. 1).

### Health impact according to COVID-19 clinical status

The physical and mental health was compared among individuals according to COVID-19 clinical status: those with COVID-19 symptoms with no test done or those with a positive COVID-19 test result (Group 1); and individuals with no test done and no COVID-19 symptoms or those with a negative COVID-19 test result (Group 2);

Results showed that Group 1 reported worse both physical (*χ*^2^ = 7.41; *V*_*c*_ = 0.074; *p* = 0.025) and mental (*χ*^2^ = 8.00; *V*_*c*_ =  = 0.077; *p* = 0.024) health than before the pandemic; a worse health self-perception than 1 year ago (*χ*^2^ = 22.95; *V*_*c*_ = 0.077; *p* = 0.002); and increased disability (mean/SD) (Group 1, 8.17/8.5; Group 217.30/6.5; *p* = 0.002) than Group 2.

For mental health, there were statistically significant differences in MDE (*χ*^2^ = 26.24; *V*_*c*_ = 0.143; *p* = 0.002), GAD (*χ*^2^ = 13.23; *V*_*c*_ = 0.219; *p* = 0.006) and STB (*χ*^2^ = 29.05; *V*_*c*_ = 0.128; *p* = 0.002). Specifically, new cases (Incidence), those positive in both assessments (Persistence) and those positive but negative during pandemic (Recovery) of MDE, GAD and STB during the COVID-19 pandemic were higher in those individuals with a positive test result or COVID-19 symptoms but no test done (Group 1) comparing with those with a negative test result or with no COVID-19 symptoms and no test done (Group 2) (Table 3). Finally, we assessed the symptomatology of PTSD according to COVID-19 status. A non-parametric U-Mann test analysis showed there were statistically significant differences between groups (*p* = 0.025). Group 1 had statistically higher PTSD symptoms than Group 2 (median/Q1-Q3) (Group 1, 13/5–34; Group 2, 8/3–20).

**Table 3:** Association between COVID-19 clinical status (positive or those with no test done but COVID-19-related symptoms vs. Negative test result or No test done and no COVID-19 symptoms) and physical and mental health.

	COVID-19 clinical status					
	Positive test result/no test done but COVID-19 symptoms		Negative test result/no test done and no COVID-19 symptoms		*χ*^2^	*p*
	N	%	N	%		
Physical health						
Self-perception					**7.41**	**0.025**
Excellent/very good	15	16.1	330	26.3		
Good	25	55.9	693	55.1		
Fair/poor	**26**	**28.0**	233	18.6		
Current general health self-perception 1 year ago					**22.95**	**0.002**
Much/Somewhat better	6	6.5	81	6.4		
Same	48	50.5	905	72.0		
Much/Somewhat worse	**41**	**43.0**	271	21.6		
Disability (mean/SD)	**20.17**	**8.5**	17.30	6.5		0.002*
Mental health						
Self-perception					**8.00**	**0.024**
Excellent/very good	39	41.5	551	43.9		
Good	37	39.4	579	46.1		
Fair/poor	**18**	**19.1**	125	9.9		
Major depressive episode					**26.24**	**0.002**
No	82	73.5	1555	89.2		
Incidence	**16**	**14.2**	88	5.0		
Persistence	5	4.4	38	2.2		
Recovery	9	8.0	63	3.6		
Generalized anxiety disorder					**13.23**	**0.006**
No	13	41.9	196	68.9		
Incidence	7	25.8	58	20.1		
Persistence	**5**	**16.1**	16	5.7		
Recovery	**5**	**16.1**	15	5.3		
Any suicidal thoughts and behaviors					**29.05**	**0.002**
No	70	67.6	1451	86.7		
Incidence	5	**4.8**	22	1.9		
Persistence	10	**9.5**	65	3.9		
Recovery	19	**18.1**	126	7.5		
Posttraumatic stress disorder symptoms (median/ Q1–Q3)	13	5–34	8	3–20		0.025**

Q_1_, First quartile_;_ Q_3_, Third quartile.

% weighted follow-up sample weight (inverse probability weighting and post-stratification).

*Student’s t-test for independent samples; ** U-Mann non-parametric test for independent samples.

Significant values are in bold.

### Sensitivity analyses

Sensitivity analyses conducted after collapsing physical and mental health outcomes and COVID-19 symptoms, significance was maintained in all statistical analyses performed (Supplementary Table S2) suggesting that our results are consistent across groups.

## Discussion

### Main results

This study explored the impact in adults over the nine months following the start of the first lockdown response to the COVID-19 pandemic in Spain. Results show there was a substantial impact on physical and mental health in the Spanish population. Specifically, in physical health, individuals reported they had lost weight, but there were no qualitatively substantial changes in BMI; fewer occasional and current smokers; but not a worsening in disability. As for mental health, there was a worsening in mental health self-assessment; a statistically significant higher prevalence of MDE and GAD, but a lower STB. The general Spanish population was mostly affected by GAD, with 1 out of 5 people defining as an incident case, and 6.8% persisting in GAD in both assessments. For MDE, our study population had a MDE incidence rate of 5.3/100 and 3.9% showed MDE before and during the pandemic. The highest percentage of individuals recovering from all mental health problems was for STB, where the prevalence was lower during the pandemic than before.

When we compared mental health status according to COVID-19 symptoms and diagnosis, individuals who had been diagnosed with COVID-19 or who had compatible symptoms had worse self-assessment of their physical and mental health and more disability than before the pandemic. For mental health, individuals who had been diagnosed with COVID-19 had more incidence, persistence and recovery of MDE, GAD and STB. In PTSD, we found that those with greater symptoms were those with related COVID-19 symptoms but who had had no test done and for those with a diagnosis of COVID-19.

### Strengths and limitations

The results of this study have to be seen in the light of its limitations. First, the attrition rate (53.1%) at follow-up might have biased comparisons between our baseline results of the total Spanish population. We addressed these limitations by adding an extended additional sample of individuals with similar characteristics to those who did not respond at follow-up. Furthermore, we applied population-based adjustments and inverse-probability weighting to correct for bias in the comparisons, which proved to be an effective method for reducing bias from a lack of response^28,29^. Secondly, the assessment of mental disorders and suicide risk was based on self-reports and not on direct clinical assessment. Therefore, they would be better considered as “probable cases” of disorder. Nevertheless, good diagnostic agreement was reported with the clinical judgment of the CIDI instrument, which includes an assessment of MDE and STB in our study, with face-to-face^20,30^, and online assessments^31^ in the Spanish population. However, although the GAD-7 and PCL-5 are well-validated scales^21,23^, calibration studies have not been carried out on general population samples. Furthermore, some scales were developed ad hoc for this study, such as the COVID-19 survey, so their diagnostic properties for detecting cases are not available. The urgency of the pandemic situation and the necessity of developing our study to add new knowledge about the impact of the pandemic and the social restrictions during the first year of pandemic motivated us to develop these scales without studying their validity. Thirdly, due to the infrequency of some variables, we had to combine information to avoid numerical problems in statistical analyses and we did sensitivity analyses with collapsed variables for measuring consistency. So, it was not possible to analyse them separately, due to the low frequency of these outcomes. Fourthly, we assessed PTSD symptoms instead of PTSD disorder. So, we do not have the mechanisms for diagnosis, and it is not possible to estimate the prevalence of PTSD in our population. However, although the DSM-5 definition notes that a life-threatening illness or debilitating medical condition is not necessarily a traumatic event. Since the first case of contagion appeared, more than 6 million people died by COVID-19 according to WHO and much more people had sequelae after the infection^32^. Therefore, although many people were not mental health affected by the consequences of COVID-19 pandemic and contagion, it is true that a subgroup of our sample have suffered some type of trauma related, especially in high-risk groups.

Nevertheless, the study has a number of strengths. First, the prospective design, with an assessment before and during the first months of the COVID-19 pandemic over a wide range of health outcomes, especially in mental health, provides a more comprehensive view of the impact of the pandemic on the health of the general Spanish population, and in people diagnosed with, or showing symptoms of, COVID-19. The longitudinal design allowed us to assess the trajectories (number, incidence, persistence, recovery) of many variables of interest. Second, we analysed two mental disorders (MDE, GAD), symptoms of PTSD and STB using adapted and validated instruments. Finally, the online methodology used tends to deliver more reliable information about sensitive information, such as suicide risk, than face-to-face assessments^33^.

### Comparison with other studies

Results showed there were an impact on physical health, with BMI having reduced significantly; although, the difference was only 2 kg. Thus, we consider this change as not clinically relevant. Furthermore, there were no qualitative changes in BMI which is in line with this hypothesis. More important, is that the general health self-assessment was worsened during the pandemic. The lockdown and further restrictions in the first year are having an impact on the general population with an increasing in Disability-Adjusted Life Years^34^; and many of those experiencing disability did not receive care, due to the closure of outpatient services or an increase in waiting lists^35^.

Our study shows a significant increase in the prevalence of mental health disorders during the pandemic in the Spanish population, from 6.5 to 8.8% in MDE, and from 13.7 to 17.7% in GAD. In two recent meta-analyses of the mental impact of COVID-19 worldwide, both showed a higher prevalence of depression (26% and 16%, respectively) than our study; a similar prevalence of anxiety (15%) was seen in only one meta-analysis, while another meta-analysis showed a higher prevalence of anxiety (32%)^36,37^. These disparities suggest that the results should be interpreted with caution, because much heterogeneity exists between studies. We used online diagnostic instruments, but most of the studies included in the meta-analyses used scales which pooled results may have overestimated. Also, most of the samples were from China, so these results may not extend well to the Spanish or European population; also, the impact of the pandemic in the population may be different for studies occurring during and after the lockdown. In Spain, two previous population-based, cross-sectional studies^38,39^ showed that the prevalence of depressive symptoms was 19% and 24%, and anxiety symptoms was 22% and 26% during the first wave. While these values are higher than our findings, it must be noted that those studies did not use diagnostic-oriented tools, but a screening instrument assessment.

Longitudinal studies with assessments before and during the pandemic showed an increase in depressive symptoms in adolescents from Iceland^40^. In adults, the British population had an increase in depressive and anxiety symptoms in the early stages of lockdown, which declined fairly rapidly; possibly because individuals adapted to the circumstances^41^. In The Netherlands, only individuals without previous mental disorders showed an increase in depressive and anxiety symptoms, but not individuals with no previous mental disorders^11^. Finally, a Spanish cohort sample assessed the prevalence of MDE in the first wave using CIDI, which was the same diagnostic instrument as our study but contacts were made by telephone and an abbreviated version was used; this increased from 7.8 to 9.8% in May/June 2020, but was not statistically significant^13^. When comparing results, our study shows that that the prevalence of MDE was significantly higher. This result suggests that the impact of the pandemic on mood increased over time more than just during the lockdown.

We also assessed the impact of the pandemic for STB in the general population and the results showed a significant decrease in STBs from 15.1 to 7.1%. These results are in line with previous studies. The evidence showed a decrease in suicide rates compared with the expected number in 12 countries from 21, and no country showed a significant increase in suicides. Specifically in Spain, the rates of suicides have reduced by 23% compared to before the pandemic^42^. To the best of our best knowledge, two population-based cross-sectional studies assessed STBs in Spaniards. In one study, the 30-day prevalence of STB was 4.5%^43^, lower than in our study. However, we assessed the presence of STBs from when the lockdown started (9 months later). Another study assessed passive suicide ideation in March 2020, just at the beginning of the lockdown. Results showed a prevalence of 8.8% for passive suicide ideation, which was lower than in our study (11.3%)^44^. In the aforementioned Spanish longitudinal study^13^, the prevalence of suicidal ideation was quite similar (2.2% vs. 2.1%). So, although some risk factors are increasing (e.g., MDE and GAD), the prevalence of STB was decreasing during the first year of the pandemic. The lack of increase in suicides and STB since the pandemic started could be attributed to the presence of protective factors or attrition rates in this specific subgroup. Communities might have actively tried to support at-risk individuals, people might have connected in new ways and some relationships might have been strengthened by households spending more time with each other^45^. For some people, the collective feeling of “we’re all in this together” might have been beneficial^42^. Further research should assess whether or not there is an increase in STB and suicide rates in the population in the long term, as the exposure of some risk factors for suicide are increasing.

Finally, we assessed mental disorder trajectories and COVID-19 status. Those individuals diagnosed with COVID-19 or with compatible symptoms were the most affected mentally by MDE, GAD, STB and PTSD symptoms. These are in line with previous studies, where the prevalence of depression^36^, anxiety^36^, PTSD^46^ and suicidal ideation^47^ were high (55% for depression; 56% anxiety; 28% PTSD, and 12% suicidal ideation) and substantially higher than in the general population. The psychiatric consequences of SARS-CoV-2 infection can be caused both by the immune response to coronaviruses, which induces local and systemic production of cytokines, chemokines and other inflammatory mediators^48^, or by psychological stressors, such as social isolation, the psychological impact of a severe and potentially fatal novel illness, concerns about infecting others and stigma^46^.

As a conclusion, although the initial effect of the pandemic in its first year has been moderate regarding physical and mental health, many risk factors have increased. Incidence, persistence and recovery of MDE, GAD and STB; the presence of PTSD symptoms in those diagnosed with COVID-19 or with compatible symptoms; and a worsening in self-assessed health status in the general population are reasons for concern. Many of these increases are regarded as known suicide risk factors. So, we expect a constant increase in mental disorders and STB in our population. This suggests the development is needed of a broad, population-based prevention approach to help people cope with the consequences of the pandemic. Such an approach should be all-encompassing, including financial measures, while also reducing the physical and mental health impact of COVID-19. Future research should gather information about the long-term impact of the pandemic beyond its initial impact, and the trajectories of some vulnerable groups, such as those with previous psychiatric disorders or those with socio-economic difficulties. Additionally, it would be useful to get the exact timing of the onset or recovery from each mental disorder or STB.

## Supplementary Information

Supplementary Information.
