# Supplementary material for: Physical and mental health impact of the COVID-19 pandemic at first year in a Spanish adult cohort
Source: Sci Rep. 2023 Mar 20;13:4547. doi: 10.1038/s41598-023-28336-2 (PMC10026238; doi:10.1038/s41598-023-28336-2)
Supplement: Supplementary file 1 — Supplementary Information. [file 41598_2023_28336_MOESM1_ESM.docx]

**Supplementary Table S1.** Weighted socio-demographic differences of total sample at baseline (n= 2,005) and the subsample at follow-up (n= 1,357).

| **Socio-demographic variables** | **Baseline (n=2,005)** | | **Follow-up (n= 1,357)** | |
| --- | --- | --- | --- | --- |
|  | **N** | **%** | **N** | **%** |
| Sex, *(men)* | 969 | 48.5 | 824 | 44.0 |
| Age, *(years)*  18-25  >25-40  >40-65  >65 | 125  444  1,074  355 | 6.3  22.2  53.8  17.8 | 85  323  1,076  390 | 4.5  17.3  57.4  20.8 |

| Marital status  Single  Married/Couple  Separated  Divorced  Widowed | 625  1,130  23  120  100 | 31.3  56.6  1.1  6.0  5.0 | 395  792  22  73  68 | 30.4  58.1  1.6  5.1  4.8 |
| --- | --- | --- | --- | --- |
| Employment status  Employed  Off sick  Unemployed  Homemaker  Student  Student & employed  Temporal or permanent disability  Retired | 1,090  30  158  139  71  57  44  408 | 54.6  1.5  7.9  7.0  3.6  2.8  2.2  20.4 | 722  14  107  79  58  29  31  310 | 53.3  1.0  8.0  5.4  4.7  2.4  2.3  22.9 |

| Region  Andalucía  Aragón  Asturias  Baleares  Cantabria  Castilla La Mancha  Castilla y León  Cataluña  Extremadura  Galicia  Islas Canarias  La Rioja  Madrid  Murcia  Navarra  Comunidad Valenciana  País Vasco | 899  125  115  90  63  211  266  709  114  317  69  30  609  118  41  422  197 | 20.5  2.8  2.6  2.0  1.4  4.8  6.1  16.1  2.6  7.2  1.6  0.7  13.9  2.7  0.9  9.6  4.5 | 342  55  50  47  17  112  96  345  49  114  43  15  243  58  21  184  83 | 18.2  2.9  2.7  2.5  0.9  6.0  5.1  18.4  2.6  6.1  2.3  0.8  13.0  3.1  1.1  9.8  4.4 |
| --- | --- | --- | --- | --- |

N= number. * % weighted follow-up sample with inverse probability weighting.

**Supplementary Table S2.** Sensitivity analyses between COVID-19 clinical status (positive or those with no test done but COVID-19-related symptoms vs. Negative test result or No test done and no COVID-19 symptoms) and physical and mental health after collapsing variables.

|  | **COVID-19 clinical status** | | | |  |  |
| --- | --- | --- | --- | --- | --- | --- |
|  | **Positive test result / no test done but COVID-19 symptoms** | | **Negative test result / no test done and no COVID-19 symptoms** | | ***χ^2^*** | **p** |
|  | **N** | **%** | **N** | **%** |  |  |
| *Physical health* |  |  |  |  |  |  |
| Self-perception |  |  |  |  | 4.31 | 0.038 |
| Excellent/Very good/Good | 40 | 72.0 | 1,032 | 81.4 |  |  |
| Fair/Poor | 26 | 28.0 | 233 | 18.6 |  |  |
| Current general health self-perception 1 year ago |  |  |  |  | 23.46 | <0.001 |
| Much/Somewhat better/Same | 54 | 57.0 | 986 | 78.4 |  |  |
| Much/Somewhat worse | 41 | 43.0 | 271 | 21.6 |  |  |
| *Mental health* |  |  |  |  |  |  |
| Self-perception |  |  |  |  | 7.81 | 0.005 |
| Excellent/Very good/Good | 96 | 80.9 | 1,130 | 90.0 |  |  |
| Fair/Poor | 18 | 19.1 | 125 | 9.9 |  |  |
| Major Depressive Episode |  |  |  |  | 27.46 | <0.001 |
| No | 82 | 73.5 | 1,555 | 89.2 |  |  |
| Incidence/Persistence/Recovery | 30 | 26.6 | 189 | 10.8 |  |  |
| Generalized Anxiety Disorder |  |  |  |  | 7.96 | 0.005 |
| No | 13 | 41.9 | 196 | 68.9 |  |  |
| Incidence/Persistence/Recovery | 17 | 58.0 | 89 | 31.1 |  |  |
| Any suicidal thoughts and behaviors |  |  |  |  | 29.28 | <0.001 |
| No | 70 | 67.6 | 1,451 | 86.7 |  |  |
| Incidence/Persistence/Recovery | 34 | 32.4 | 213 | 13.3 |  |  |

N= number. % weighted follow-up sample with inverse probability weighting.
